# Quantifying the effects of long-range ^13^C-^13^C dipolar coupling on measured relaxation rates in RNA

**DOI:** 10.1007/s10858-021-00368-8

**Published:** 2021-04-29

**Authors:** Lukasz T. Olenginski, Theodore K. Dayie

**Affiliations:** Department of Chemistry and Biochemistry, Center for Biomolecular Structure and Organization, University of Maryland, College Park, MD 20742 USA

**Keywords:** Dipolar coupling, Relaxation, Dynamics, NMR spectroscopy, Nucleic acids

## Abstract

Selective stable isotope labeling has transformed structural and dynamics analysis of RNA by NMR spectroscopy. These methods can remove ^13^C-^13^C dipolar couplings that complicate ^13^C relaxation analyses. While these phenomena are well documented for sites with adjacent ^13^C nuclei (e.g. ribose C1′), less is known about so-called isolated sites (e.g. adenosine C2). To investigate and quantify the effects of long-range (> 2 Å) ^13^C-^13^C dipolar interactions on RNA dynamics, we simulated adenosine C2 relaxation rates in uniformly [U-^13^C/^15^N]-ATP or selectively [2-^13^C]-ATP labeled RNAs. Our simulations predict non-negligible ^13^C-^13^C dipolar contributions from adenosine C4, C5, and C6 to C2 longitudinal (R_1_) relaxation rates in [U-^13^C/^15^N]-ATP labeled RNAs. Moreover, these contributions increase at higher magnetic fields and molecular weights to introduce discrepancies that exceed 50%. This will become increasingly important at GHz fields. Experimental R_1_ measurements in the 61 nucleotide human hepatitis B virus encapsidation signal ε RNA labeled with [U-^13^C/^15^N]-ATP or [2-^13^C]-ATP corroborate these simulations. Thus, in the absence of selectively labeled samples, long-range ^13^C-^13^C dipolar contributions must be explicitly taken into account when interpreting adenosine C2 R_1_ rates in terms of motional models for large RNAs.

**Supplementary Information:**

The online version contains supplementary material available at 10.1007/s10858-021-00368-8.

## Introduction

RNAs are important macromolecules that function in a wide range of cellular roles (Cech and Steitz 2014; Mortimer et al. 2014; Sharp 2009). Despite being composed of only four ribonucleotide building blocks, RNAs are capable of adopting complex three-dimensional structures that impart functionality (Dethoff et al. 2012; Ganser et al. 2019). Moreover, RNAs are dynamic and can sample numerous conformations on various time scales that might be important for function (Zhao et al. 2017; Marušič et al. 2019). Solution nuclear magnetic resonance (NMR) spectroscopy is a high-resolution biophysical technique that is well suited to probe these dynamic RNA structures. Three commonly measured dynamics parameters are the longitudinal (R_1_) and transverse (R_2_) relaxation rates and the heteronuclear Overhauser effect (hNOE) (Marušič et al. 2019; Palmer 2004; Wagner 1993). The R_1_ rate measures the return of the longitudinal magnetization to thermal equilibrium whereas R_2_ measures the decay of transverse magnetization, and the hNOE measures the change in heteronuclear spin magnetization in response to saturating proton spins (Yamazaki et al. 1994; Peng and Wagner 1992; Abragam 1961).

RNA motions directly influence these R_1_, R_2_, and hNOE relaxation parameters (Marušič et al. 2019; Palmer 2004; Wagner 1993; Yamazaki et al. 1994; Peng and Wagner 1992; Abragam 1961; Spiess 1978; Hansen and Al-Hashimi 2007; Nirmala and Wagner 1988; Palmer et al. 1991). Depending on the probed nuclei, dipolar interactions and chemical shielding anisotropy (CSA) mechanisms contribute predominantly to R_1_ and R_2_ relaxation, and dipolar interactions to the hNOE. These three relaxation measurements can be fit to extract motional variables such as overall correlation time (${\tau }_{\mathrm{C}}$) and the square of the generalized order parameter (S^2^) that describe fast (ps-ns) dynamics in RNA within a Model Free formalism (Spiess 1978; Lipari and Szabo 1982) or combined Model Free and reduced spectral density mapping implemented in ROTDIF (Berlin et al. 2013). Therefore, accurate R_1_, R_2_, and hNOE measurements are crucial for obtaining precise dynamics information and drawing valid conclusions about RNA molecular recognition events.

These dynamic RNA motions are most often measured by using ^13^C (Boisbouvier et al. 1999; Dayie et al. 2002) and ^15^N (Dayie et al. 2002; Akke et al. 1997) nuclei as indirect probes. However, imino and amino ^15^N nuclei experience solvent exchange and are only visible in structured regions, making non-protonated ^15^N (e.g. purine N7) and protonated ^13^C (e.g. adenosine C2) nuclei attractive probes. The latter sites are present in both the nucleobase and ribose moieties and therefore provide more coverage of RNA structure. Still, large ^13^C-^13^C scalar and dipolar couplings in uniformly ^13^C/^15^N labeled RNA can complicate analyses of these sites (Yamazaki et al. 1994; Kay et al. 1989; Alvarado 2014; Johnson et al. 2006; Thakur and Dayie 2012; Nam et al. 2020; Thakur et al. 2012). Indeed, the effect of dipolar couplings on RNA relaxation has been studied for sites with adjacent ^13^C nuclei (e.g. ribose C1′). These investigations demonstrate that dipolar interactions from attached ^13^C atom(s) lead to deviations from monoexponential decay and discrepancies in the extracted R_1_ rate for ribose C1′ (Alvarado 2014; Thakur and Dayie 2012; Nam et al. 2020; Thakur et al. 2012), C2′ and C4′ (Johnson et al. 2006), as well as for pyrimidine C6 (Nam et al. 2020; Thakur et al. 2012). However, much less is known about isolated sites such as purine C8 or adenosine C2. We recently showed that purine C8 may experience non-negligible dipolar contributions to R_1_ relaxation from non-adjacent coupling partners (Nam et al. 2020). The extent to which adenosine H2-C2 approximates an isolated spin pair remains unclear.

To investigate and quantify the effects of long-range (> 2 Å) ^13^C-^13^C dipolar coupling on RNA dynamics, we simulated adenosine C2 relaxation rates in uniformly [U-^13^C/^15^N]-ATP or selectively [2-^13^C]-ATP labeled RNAs. Our simulations predict non-negligible ^13^C-^13^C dipolar contributions from adenosine C4, C5, and C6 to C2 R_1_ rates in [U-^13^C/^15^N]-ATP labeled RNAs that increase with higher magnetic fields and molecular weights. To empirically test our simulations, we measured adenosine C2 relaxation in the 61 nucleotide (nt) human hepatitis B virus encapsidation signal ε (HBV ε) RNA (Flodell et al. 2006; Lee 1997; Knaus and Nassal 1993; Hirsch et al. 1990) labeled with [U-^13^C/^15^N]-ATP or [2-^13^C]-ATP. To this end, we used our recently synthesized [2-^13^C, 7-^15^N]-ATP (Olenginski and Dayie 2020) as a selective adenosine ^13^C2 labeled probe. We demonstrate that the removal of long-range ^13^C-^13^C dipolar coupling partners reveals discrepancies in measured adenosine C2 R_1_ values between uniformly and selectively labeled samples. Moreover, R_1_ measurements at lower temperature (mimicking increased molecular weight) revealed exacerbated R_1,C2_ discrepancies, which further corroborates our simulations and argues that selective ^13^C2 labeled probes obviates the need to account for the significant contributions to meausured R_1,C2_ that arise from neighboring ^13^C atom(s) in RNAs with a > ${\tau }_{\mathrm{C}}$20 ns. Our [2-^13^C, 7-^15^N]-ATP (Olenginski and Dayie 2020) also showed better spectroscopic properties than [U-^13^C/^15^N]-ATP, providing and additional advantage to using selectively labeled samples to measure RNA dynamics.

## Materials and methods

### Theoretical simulations

^13^C R_1_ and R_2_ relaxation rates and steady-state ^13^C{^1^H} hNOE values were simulated using Eqs. 1–6, (Palmer 2004; Peng and Wagner 1992; Abragam 1961). We assumed isotropic tumbling. These relaxation parameters were simulated for adenosine C2 in [U-^13^C/^15^N]-ATP or [2-^13^C]-ATP labeled RNAs and included dipolar contributions from adenosine C4, C5, C6, N1, and N3 at average distances of 2.20, 2.70, 2.30, 1.40, and 1.30 Å, respectively. In addition, adenosine C2 experiences dipolar contributions with the following protons: (1) the attached H2, (2) those within the same nucleotide, (3) those 3′-same strand, and (4) those 3′-cross-strand contacts (Wijmenga and Buuren 1998). Protons in (2) are H1′ and H2′ at average distances of 4.35 and 4.20 Å. Protons in (3) are H1, H2, amino (N)H_2_, and H1′ at average distances of 4.00, 4.40, 4.33, and 4.45 Å. Protons in (4) are H1, H3, amino (N)H_2_, and H1′ at average distances of 4.10, 4.50, 4.40, and 4.95 Å. All proton distances above were calculated from pdb 2ixy (Flodell et al. 2006) as a representative A-helical RNA. Solution NMR derived CSA values (*σ*_*11*_ = 89, *σ*_*22*_ = 15, *σ*_*33*_ = − 104) (Ying et al. 2006) and an aromatic CH bond length of 1.104 Å (Fiala et al. 2000) were used in these simulations.

### RNA sample preparation

In vitro transcriptions of RNA were performed as previously described (Milligan and Uhlenbeck 1989). In brief, transcriptions were carried out in 40 mM Tris–HCl pH 8 (at 37 °C), 1 mM spermidine, 0.01% Triton X-100, 80 mg/ml PEG, 0.3 μM DNA template, 1 mM 1,4-dithiothreitol, 2 U/μl thermostable inorganic pyrophosphatase, 5–20 mM rNTPs, 5–20 mM MgCl_2_, and 0.1 mg/mL T7 RNA polymerase. Reactions proceeded for 3 h at 37 °C. For each RNA transcribed, the concentrations of MgCl_2_ and rNTPs were optimized. DNA template and [U-^13^C/^15^N]-ATP were purchased from Integrated DNA Technologies and Cambridge Isotope Laboratories, respectively. The [2-^13^C, 7-^15^N]-ATP was synthesized as recently described (Olenginski and Dayie 2020). After transcription, samples were extracted with acid-phenol:chloroform, ethanol precipitated, purified by preparative denaturing gel electrophoresis, and electroeluted. The samples were subsequently dialyzed five times against UltraPure ddH_2_O, folded in NMR buffer (10 mM Na_3_PO_4_ pH 6.5, 0.1 mM EDTA), lyophilized, and resuspended in 100% D_2_O. NMR samples of HBV ε labeled with [U-^13^C/^15^N]-ATP or [2-^13^C, 7-^15^N]-ATP had a final concentration of 0.35 mM in 0.30 ml (calculated using a molar extinction coefficient of 768.3 mM^−1^ cm^−1^).

### NMR spectroscopy

All NMR experiments were performed on an 800 MHz Avance III Bruker spectrometer equipped with a triple resonance cryogenic probe. NMR relaxation data were collected at either 5 or 25 °C as specified in the text and figure legends. TROSY-detected measurements of ^13^C R_1_ and R_1ρ_ relaxation rates and steady-state ^13^C{^1^H} hNOE values were adapted from previous pulse sequences (Hansen and Al-Hashimi 2007; Lakomek et al. 2013). For R_1_ experiments at 25 °C, relaxation delays of 0.10, 0.20 (× 2), 0.36, 0.50, 0.90, and 1.20 s (with both [U-^13^C/^15^N]-ATP and [2-^13^C, 7-^15^N]-ATP labeled sample) were used. For R_1_ experiments at 5 °C, relaxation delays of 0.10, 0.20 (× 2), 0.80, 1.00, 1.20 (with [U-^13^C/^15^N]-ATP labeled sample) or 0.10, 0.20 (× 2), 0.90, 1.10, 1.30 (with [2-^13^C, 7-^15^N]-ATP labeled sample) were used. For R_1ρ_ experiments, relaxation delays of 1.5, 2.4, 3.4, 4.6, 6.1, 8.0, and 11.0 ms (at 25 °C) or 1.0, 2.0, 4.0, 5.0, and 6.0 ms (at 5 °C) were used and the strength of the spin-lock field (ω_1_) was 1.9 kHz. R_1_ and R_1ρ_ experiments were acquired in an interleaved manner as a pseudo-three-dimensional experiment and using a recycle delay of 2.5 s. For ^13^C{^1^H} hNOE (saturation) experiments, recycle and saturation delays of 1.5 and 7 s were used and proton saturation was achieved using a train of hard 180° pulses. In the ^13^C{^1^H} hNOE (no saturation) experiments, a delay of 8.5 s was used in order to match the time of both recycle and saturation delays from the saturation experiment. For experiments on [U-^13^C/^15^N]-ATP labeled HBV ε, selective pulses were applied as previously described (Hansen and Al-Hashimi 2007; Nam et al. 2020). Shape pulses used for on-resonance ^13^C inversion, on-resonance ^13^C refocusing, and off-resonance ^13^C inversion were Q3 (Emsley and Bodenhausen 1992), RSNOB (Kupče et al. 1995), and IBURP2 (Geen and Freeman 1991), respectively. Q3 pulse selectively inverts the ^13^C magnetization of interest, whereas RSNOB and IBURP2 selectively refocus (invert) ^13^C magnetization to eliminate ^13^C-^13^C scalar coupling evolution. Pulse lengths for each pulse were 937.5, 1000, and 450 μs, respectively. The offset and bandwidth for IBURP2 were − 40 and 50 ppm, respectively. ^15^N was decoupled.

### Data analysis

NMR spectra were processed and analyzed using TopSpin 4.0, NMRFx Processor, and NMRViewJ (Norris et al. 2016; Johnson and Blevins 1994). R_1_ and R_1ρ_ relaxation rates were determined by fitting peak intensities to a monoexponential decay. Uncertainties in R_1_ rates were estimated by propagating the error in peak intensities from duplicated delay points (indicated by “ × 2” above). R_2_ rates were corrected for the off-resonance ω_1_ using Eqs. 7 and 8. Uncertainties in R_1ρ_ rates were determined by the RELAXFIT (Fushman et al. 1997) Matlab program. The steady-state ^13^C{^1^H} hNOE was obtained using (1 + η) (Peng and Wagner 1992; Palmer et al. 1991; Clore et al. 1990a, b; Weaver et al. 1988). Uncertainties in ^13^C{^1^H} hNOE values were estimated by propagating the error in peak intensities in duplicated experiments.

## Results and discussion

### Effects of long-range dipolar couplings on adenosine C2 relaxation

Before quantifying the effects of dipolar couplings on RNA dynamics, it is informative to consider the various relaxation contributions to our targeted nuclei. The ^13^C R_1_ and R_2_ rates of adenosine C2 (R_1,C2_ and R_2,C2_) are given by

1 $${R}_{1,C2}=\sum_{i}{R}_{1,C2,{H}_{i}}+\sum_{j}{R}_{1,C2,{C}_{j}}+\sum_{k}{R}_{1,C2,{N}_{k}}+{R}_{1,CSA}$$

2 $${R}_{2,C2}=\sum_{i}{R}_{2,C2,{H}_{i}}+\sum_{j}{R}_{2,C2,{C}_{j}}+\sum_{k}{R}_{2,C2,{N}_{k}}+{R}_{2,CSA}+{R}_{ex},$$

wherein the auto R_1,C2_ (${R}_{C}\left({C}_{z}\right)$) and R_2,C2_ (${R}_{C}\left({C}_{x,y}\right)$) rates and cross-relaxation (${R}_{C}\left({{H}_{z}^{C}\to C}_{z}\right)$) are functions of the underlying spectral density function (Palmer 2004; Peng and Wagner 1992; Abragam 1961):

3 $${R}_{1,C2}={R}_{C}\left({C}_{z}\right)=\frac{1}{4}{{ D}_{C,i}}^{2}\left[J\left({\omega }_{i}-{\omega }_{C}\right)+3J\left({\omega }_{C}\right)+6J\left({\omega }_{C}+{\omega }_{i}\right)\right]+{{C}_{C}}^{2}\left[J\left({\omega }_{C}\right)\right]$$

4 $${R}_{C}\left({{H}_{z}^{C}\to C}_{z}\right)=\frac{1}{4}{{D}_{C,i}}^{2}\left[6J\left({\omega }_{C}+{\omega }_{i}\right)-J\left({\omega }_{i}-{\omega }_{C}\right)\right]$$

5 $${R}_{2,C2}={R}_{C}\left({C}_{x,y}\right)=\frac{1}{8}{{D}_{C,i}}^{2}\left[4J\left(0\right)+J\left({\omega }_{i}-{\omega }_{C}\right)+3J\left({\omega }_{C}\right)+6J{(\omega }_{i})+6J\left({\omega }_{C}+{\omega }_{i}\right)\right]+\frac{1}{6}{{C}_{C}}^{2}\left[4J\left(0\right)+3J\left({\omega }_{C}\right)\right]$$

R_ex_ is the chemical exchange contribution to R_2_, D_C,i_ and C_C_ are the dipolar coupling (${\mu }_{0}{\gamma }_{C}{\gamma }_{i}\hslash$/$4\uppi {r}^{3}$) and CSA (${\omega }_{C}\Delta {\sigma }_{C}/\sqrt{3}$) constants, respectively, where ${\gamma }_{i}$ is the gyromagnetic ratio of spin i (where i can be ^1^H, ^13^C, or ^15^N), r is the distance between the two spins, ${\mu }_{0}$ is the permeability of free space, $\hslash$ is Plank’s constant divided by 2p, and $\Delta {\sigma }_{C}= \sqrt{\left({\sigma }_{x}^{2} +{\sigma }_{y}^{2}- {\sigma }_{x}{\sigma }_{y}\right)}$. Here, *σ*_*x*_ = *σ*_*33*_ − *σ*_*11*_, *σ*_*y*_ = *σ*_*33*_ − *σ*_*22*_ and *σ*_*11*_, *σ*_*22*_, and *σ*_*33*_ are the principal components of the chemical shielding tensor (Ying et al. 2006; Fushman et al. 1998) and $J(\omega )$ is the spectral density function assuming isotropic tumbling. The auto- and cross-relaxation rates combine to give the steady-state ^13^C{^1^H} hNOE (η) (Peng and Wagner 1992; Palmer et al. 1991; Clore et al. 1990a, b; Weaver et al. 1988)

6 $$\eta = \left( {\frac{{I_{{{\text{sat}}}} {-} I_{{{\text{eq}}}} }}{{I_{{{\text{eq}}}} }}} \right) = \frac{{\gamma_{{\text{H}}} R_{{\text{C}}} \left( {H_{{\text{z}}}^{{\text{C}}} \to C_{{\text{z}}} } \right)}}{{\gamma_{{\text{C}}} R_{{1,{\text{C}}2}} }},$$

where *I*_sat_ and *I*_eq_ are signal intensities of the ^13^C resonances when the ^1^H resonances are saturated or not.

As seen in Eqs. 1–5, adenosine R_1,C2_ and R_2,C2_ rates incorporate dipolar interactions with nearby ^1^H, ^13^C, and ^15^N nuclei, as well as the ^13^C CSA. For small-to-medium sized RNAs with a ${\tau }_{\mathrm{C}}$ < 10 ns, ^1^H-^13^C dipolar and ^13^C CSA contributions dominate adenosine R_1,C2_ and R_2,C2_ relaxation. At 800 MHz, the ^1^H-^13^C dipolar and ^13^C CSA contribution to adenosine R_1,C2_ is 43–71% and 29–52%, respectively, whereas their contribution to adenosine R_2,C2_ is 43–64% and 36–57%, respectively (^13^C CSA and dipolar contributions are defined as [100*(^13^C2 CSA/R_1/2,C2_)] and [100*(^1^H-^13^C dipolar/R_1/2,C2_)], [100*(^13^C-^13^C dipolar/R_1/2,C2_)], or [100*(^13^C-^15^N dipolar/R_1,C2_)], respectively, where the CSA and dipolar terms are those found in Eqs. 3 and 5) (Supplementary Fig. S1a and b). Therefore, for these RNAs, the dipolar contributions from surrounding ^13^C (< 4%) and ^15^N (< 1%) nuclei are negligible and can likely be ignored. However, ^13^C-^13^C dipolar interactions contain an additional $J(0)$ term arising from $J\left({\omega }_{C2}-{\omega }_{X}\right)$, which increases as a function of ${\tau }_{\mathrm{C}}$ and magnetic field strength. Moreover, the bonafide $J(0)$ term in R_2,C2_ is pre-multiplied by 4 and is linked with the direct H2-C2 dipolar vector that is larger than any ^13^C-^13^C dipolar vector by ~ 16 due to the contribution from the gyromagnetic ratios alone. Taken together, ^13^C-^13^C dipolar interactions are expected to negligibly contribute to adenosine R_2,C2_ (< 0.1%) and significantly contribute to R_1,C2_ as RNAs increase in size and higher magnetic fields are used. Indeed, at 800 MHz, these ^13^C-^13^C dipolar contributions to adenosine R_1,C2_ rise to 20, 50, and 80% in larger RNAs with a ${\tau }_{\mathrm{C}}$ of 25, 50, and 100 ns (Supplementary Figs. S1c and d). Moreover, these contributions further increase at higher magnetic fields (Supplementary Figs. S1c and d).

**Fig. 1:**
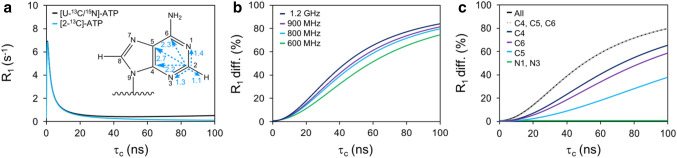
Adenosine R_1,C2_ simulations in [U-^13^C/^15^N]-ATP or [2-^13^C]-ATP labeled RNAs. **a** Simulated adenosine R_1,C2_ rates with a scheme of adenine (numbered by atom and with interatomic distances (Å) to C2) shown as an inset. **b** Simulated adenosine R_1,C2_ percent difference (diff.) [100*(R_1,C2(uniform)_ − R_1,C2(selective)_)/R_1,C2(uniform)_]. **c** Simulated adenosine R_1,C2_ percent difference for each dipolar coupling partner (C4, C5, C6, N1, and N3). All simulations assume isotropic tumbling and those in **b** were carried out with increasing magnetic field strengths and overall correlation times (${\tau }_{\mathrm{C}}$) whereas those in **a** and **c** are at 800 MHz. Solution NMR derived CSA values (*σ*_*11*_ = 89, *σ*_*22*_ = 15, *σ*_*33*_ = − 104) (Ying et al. 2006) and an aromatic CH bond length of 1.104 Å (Fiala et al. 2000) were used. Our simulations suggest that dipolar interactions result in overestimated R_1,C2(uniform)_ rates that increase with higher magnetic fields and molecular weights

In [U-^13^C/^15^N]-ATP labeled RNAs, adenosine C2 is dipolar coupled to the attached H2, the adjacent N1 and N3, and the long-range (> 2 Å) C4, C5, and C6 atoms (Fig. 1a, inset). Moreover, in fully protonated RNA, adenosine C2 also experiences long-range dipolar contributions from protons within the same nucleotide, those 3′ and on the same strand, and those 3′ and on the cross-strand (Wijmenga and Buuren 1998). If these long-range dipolar couplings contribute significantly to RNA relaxation, theoretical simulations should reveal discrepancies in adenosine R_1,C2_ and R_2,C2_ rates and steady-state ^13^C{^1^H} hNOE values in [U-^13^C/^15^N]-ATP and [2-^13^C]-ATP labeled RNAs. To test this hypothesis, we used previously reported CSA values derived from solution NMR (Ying et al. 2006) and Eqs. 1–6, (Palmer 2004; Peng and Wagner 1992; Abragam 1961) to simulate adenosine R_1,C2_ and R_2,C2_ relaxation rates and steady-state ^13^C{^1^H} hNOE values in [U-^13^C/^15^N]-ATP or [2-^13^C]-ATP labeled RNAs (see “Materials and Methods” section). For simplicity, our simulations assume isotropic tumbling as the effects of long-range dipolar couplings are more readily quantifiable.

We do not observe differences in the simulated R_2,C2_ rates or steady-state ^13^C{^1^H} hNOE values (Supplementary Fig. S2), in agreement with previous studies (Yamazaki et al. 1994; Hansen and Al-Hashimi 2007; Nam et al. 2020). However, our simulations do predict discrepancies in R_1,C2_ rates (Fig. 1a) between uniformly and selectively labeled samples (R_1,C2(uniform)_ and R_1,C2(selective)_, respectively), an observation similar to that recently reported for purine C8 sites (Nam et al. 2020). Specifically, dipolar interactions result in overestimated R_1,C2(uniform)_ rates that increase with higher magnetic fields and molecular weights (Fig. 1a), as predicted by Eq. 3 (Supplementary Fig. S1a). Moreover, the percent difference in R_1,C2_ (defined as [100*(R_1,C2(uniform)_ − R_1,C2(selective)_)/R_1,C2(uniform)_]) is predicted to be as large as 80% at 1.2 GHz and a ${\tau }_{\mathrm{C}}$ of 100 ns (Fig. 1b). While RNAs of this size are rarely probed by NMR, the simulated discrepancies are still significant for smaller RNAs. As highlighted by our simulations, ^13^C-^13^C dipolar interactions dominate the discrepancy whereas N1 and N3 have almost no effect. Moreover, the ^13^C-^13^C contributions scale with atomic distance from C2, with C4 (2.2 Å) having the greatest effect followed by C6 (2.3 Å) and then C5 (2.7 Å) (Figs. 1a, inset and c).

### Adenosine C2 R_1_ measurements in uniformly and selectively labeled RNA

**Fig. 2:**
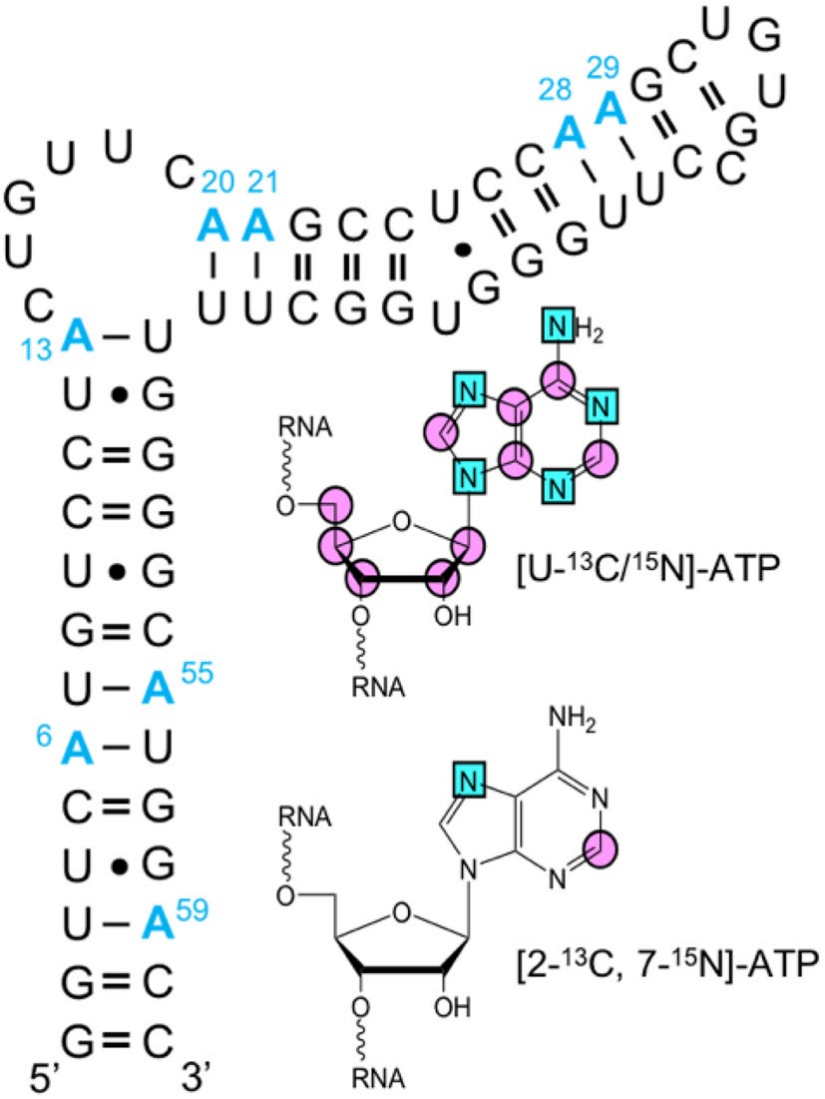
Selective and uniform RNA labeling. Sequence of the 61 nt HBV ε RNA with all [U-^13^C/^15^N]-ATP or [2-^13^C, 7-^15^N]-ATP labeled adenosine residues shown in blue and numbered. Magenta circle = ^13^C and cyan square = ^15^N

**Fig. 3:**
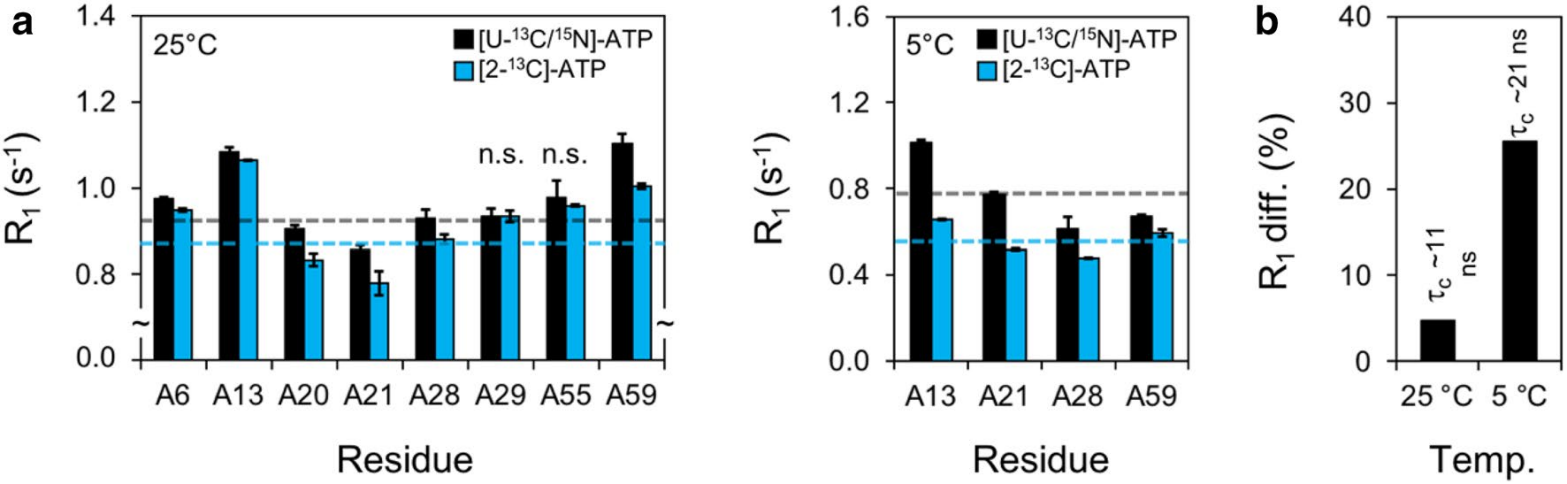
Experimental adenosine R_1,C2_ measurements in [U-^13^C/^15^N]-ATP or [2-^13^C, 7-^15^N]-ATP labeled HBV ε RNA. **a** Adenosine R_1,C2(uniform)_ and R_1,C2(selective)_ rate measurements in HBV ε at 800 MHz and 5 or 25 °C. Mean rates are shown with dashed lines and error bars represent ± standard deviation (s.d.). Experimental R_1,C2(uniform)_ rates at 25 °C were larger (outside experimental error) than R_1,C2(selective)_ for all adenosine residues except A29 and A55 (designated no significance, n.s.). Experimental R_1,C2(uniform)_ rates at 5 °C were larger than R_1,C2(selective)_ for all 4 resolved adenosine residues. **b** Average R_1,C2_ percent difference (diff.) [100*(R_1,C2(uniform)_ − R_1,C2(selective)_)/R_1,C2(uniform)_] for the data at different temperatures (temp.) shown in **a**. The average percent difference in measured R_1,C2_ rates at 25 °C was 4.7%, which agrees well with the simulated 5.4% difference for an RNA with a ${\tau }_{\mathrm{C}}$ of 11 ± 1 ns [measured from R_2_/R_1_ ratio (Fushman et al. 1994; Thakur et al. 2010)]. The average percent difference in measured R_1,C2_ rates at 5 °C was 25.5%, compared to the simulated 15.6% difference for an RNA with a ${\tau }_{\mathrm{C}}$ of 21 ± 1 ns [measured from R_2_/R_1_ ratio (Fushman et al. 1994; Thakur et al. 2010)]. Taken together, our simulations and experimental measurements suggest that the discrepancy between R_1,C2(uniform)_ and R_1,C2(selective)_ increases with higher molecular weights

Our newly synthesized [2-^13^C, 7-^15^N]-ATP (Olenginski and Dayie 2020) removes unwanted ^13^C-^13^C and ^13^C-^15^N dipolar interactions and was therefore used along with commercially available [U-^13^C/^15^N]-ATP to empirically test our simulations. To this end, we measured adenosine R_1,C2_ rates for [U-^13^C/^15^N]-ATP or [2-^13^C, 7-^15^N]-ATP labeled HBV ε (Fig. 2) at 800 MHz and 25 °C using TROSY-detected pulse sequences (Hansen and Al-Hashimi 2007; Lakomek et al. 2013; Weigelt 1998; Pervushin et al. 1997). In agreement with our simulations (Fig. 1a), R_1,C2(uniform)_ was significantly higher than R_1,C2(selective)_ for 6 of the 8 HBV ε adenosine residues (Fig. 3a). Explanations for why A29 and A55, in particular, differ from the other residues requires detailed structural information which is currently lacking. Nevertheless, our simulated and experimental trends show good agreement on the whole. That is, the average percent difference in measured R_1,C2_ rates was 4.7% (Fig. 3b) compared to the simulated 5.4% (Fig. 2b) for an RNA with a ${\tau }_{\mathrm{C}}$ of 11 ± 1 ns at 800 MHz (measured from R_2_/R_1_ (Fushman et al. 1994; Thakur et al. 2010)) (Supplementary Fig. S3). While this discrepancy is small and can likely be ignored, our simulations suggest that this is no longer true as RNAs increase in size.

To experimentally verify that the discrepancy in R_1,C2_ increases at higher molecular weights, we repeated our R_1,C2_ measurements in [U-^13^C/^15^N]-ATP or [2-^13^C, 7-^15^N]-ATP labeled HBV ε at 5 °C to simulate an RNA with a higher molecular weight (larger ${\tau }_{\mathrm{C}}$). To maximize signal-to-noise and minimize experimental time, we reduced the sweep-width and time-domain points while increasing the number of scans. Therefore, only 4 of 8 adenosine C2-H2 resonances were resolved (Supplementary Fig. S4). Nevertheless, R_1,C2(uniform)_ was again observed to be significantly higher than R_1,C2(selective)_ for all 4 resolved HBV ε adenosine residues (Fig. 3a). Moreover, the average percent difference in measured R_1,C2_ at 5 °C was significantly higher than those measured at 25 °C (Fig. 3b), in agreement with our simulations (Fig. 1b). Specifically, the average percent difference in measured R_1,C2_ rates was 25.5% (Fig. 3b), compared to the simulated 15.6% (Fig. 1b) for an RNA with a ${\tau }_{\mathrm{C}}$ of 21 ± 1 ns at 800 MHz (measured from R_2_/R_1_ (Fushman et al. 1994; Thakur et al. 2010)) (Supplementary Fig. S3).

Taken together, while relatively isolated, adenosine C2 experiences long-range ^13^C-^13^C dipolar couplings that can neither be wholly ignored nor circumvented with selective pulses in [U-^13^C/^15^N]-ATP labeled samples. That is, these dipolar contributions must be explicitly taken into account (Hansen and Al-Hashimi 2007) when interpreting adenosine R_1,C2_ rates in terms of motional models for large RNAs.

### Adenosine C2 R_1ρ_ relaxation and steady-state ^13^C{^1^H} hNOE measurements in uniformly and selectively labeled RNA

As previously described, R_1_, R_2_, and hNOE measurements are a prerequisite to a robust analysis of RNA ps-ns dynamics (Marušič et al. 2019; Palmer 2004; Wagner 1993; Lipari and Szabo 1982). We have already quantified the discrepancies that exist in adenosine R_1,C2_ measurements derived from [U-^13^C/^15^N]-ATP labeling. While we did not observe such differences in the simulated adenosine R_2,C2_ rates or steady-state ^13^C{^1^H} hNOE values (Supplementary Fig. S2), we sought to experimentally verify this for completeness. We therefore measured adenosine R_2,C2_ rates and ^13^C{^1^H} hNOE values for [U-^13^C/^15^N]-ATP or [2-^13^C, 7-^15^N]-ATP labeled HBV ε (Fig. 2) at 800 MHz and 25 °C using TROSY-detected pulse sequences (Hansen and Al-Hashimi 2007; Lakomek et al. 2013; Weigelt 1998; Pervushin et al. 1997). Observed R_1ρ_ rates contain contribution from both R_1_ and R_2_ relaxation, which are accounted for according to the relations (Hansen and Al-Hashimi 2007; Lakomek et al. 2013; Akke and Palmer 1996; Davis et al. 1994)

7 $${R}_{1\rho } ={ R}_{1}({cos}^{2}\theta ) +{ R}_{2}({sin}^{2}\theta )$$

8 $$\theta = {tan}^{-1}\left(\frac{{\omega }_{1}}{\Omega }\right).$$

Here, ${\omega }_{1}$ is the strength of the spin-lock field and $\Omega$ is the offset from the spin-lock carrier frequency. We used an R_1ρ_ experiment (Hansen and Al-Hashimi 2007; Lakomek et al. 2013; Akke and Palmer 1996; Peng et al. 1991; Korzhnev et al. 2002) to extract R_2_ rates in HBV ε. In agreement with our simulations, adenosine R_2,C2_ rates and steady-state ^13^C{^1^H} hNOE values did not differ significantly between uniformly or selectively labeled samples (Supplementary Fig. S5). For straightforward analysis, we will interpret dynamics data from our [2-^13^C, 7-^15^N]-ATP labeled sample.

**Fig. 4:**
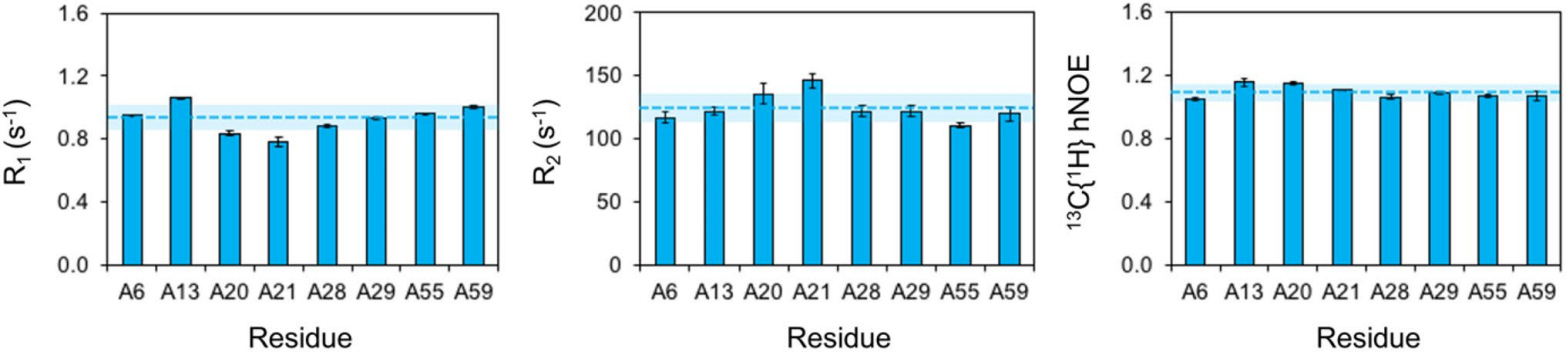
Experimental adenosine C2 relaxation measurements for [2-^13^C, 7-^15^N]-ATP labeled HBV ε at 800 MHz and 25 °C. Adenosine R_1,C2_ and R_2,C2_ (calculated from R_1ρ,C2_ using Eqs. 6 and 7) rates and steady-state^13^C{^1^H} hNOE measurements are shown. Error bars represent ± s.d. and the mean relaxation parameters are shown with dashed lines with a shaded box representing ± s.d. above and below the mean. Residue A13 shows high R_1,C2_ and hNOE suggestive of increased internal motions whereas A21 has a high R_2,C2_ rate indicative of possible R_ex_

As such, adenosine R_1,C2_ and R_2,C2_ rates measured in helical regions of HBV ε were all close to the mean, except for residues A13 and A21 (Fig. 4). Specifically, residue A13 shows high R_1,C2_ and low R_2,C2_ rates suggestive of increased internal motions (Fig. 4). Residue A21, on the other hand, has a high R_2,C2_ rate indicative of possible R_ex_ contributions (Fig. 4). In addition to R_1_ and R_2_ rates, accurate measurements of steady-state ^13^C{^1^H} hNOE values can provide further information on RNA dynamics (Marušič et al. 2019; Palmer 2004; Wagner 1993; Lipari and Szabo 1982). Consistent with adenosine R_1,C2_ and R_2,C2_ rates, residue A13 shows the highest hNOE value suggestive of increased internal motions (Fig. 4). All other adenosine C2 nuclei have hNOE values close to the mean indicative of helical residues (Fig. 4). Taken together, our [2-^13^C, 7-^15^N]-ATP label simplified probing of adenosine C2 spin relaxation measurements in HBV ε without the need for selective pulses (Emsley and Bodenhausen 1992; Kupče et al. 1995; Geen and Freeman 1991) or explicit spectral density modeling with assumed models of motion (Hansen and Al-Hashimi 2007). As an added benefit, ^1^H-^13^C TROSY spectra collected on selectively labeled HBV ε showed better signal-to-noise and narrower ^1^H linewidths compared to its [U-^13^C/^15^N]-ATP counterpart (Supplementary Fig. S6).

## Conclusion

We investigated and quantified the effect of long-range ^13^C-^13^C dipolar couplings on adenosine C2 relaxation in [U-^13^C/^15^N]-ATP and [2-^13^C, 7-^15^N]-ATP labeled RNAs. Selective ^13^C-labeling of adenosine C2 removed unwanted dipolar interactions with C4, C5, and C6 found in [U-^13^C/^15^N]-ATP. Theoretical simulations and experimental measurements revealed non-negligible overestimates in adenosine R_1,C2_ rates derived from [U-^13^C/^15^N]-ATP labeled samples that increase with higher magnetic fields and molecular weights. The agreement between our experimental and simulated R_1,C2_ rates and discrepancies at 25 °C support the predictions from our simulations. Moreover, R_1,C2_ measurements at 5 °C (increased molecular weight) revealed exacerbated R_1,C2_ discrepancies, which further confirms our simulations and argues that selective ^13^C2 labeled probes simplify R_1,C2_ measurements in RNAs with a ${\tau }_{\mathrm{C}}$ > 20 ns.

It is important to note that auto-relaxation due to the ^13^C-^13^C dipolar interaction does not lead to deviation from the expected monoexponential relaxation and can be explicitely taken into account by using the appropriate spectral density function (Hansen and Al-Hashimi 2007). Therefore, elimination of these unwanted ^13^C-^13^C dipolar contributions permits the use of simple spectral density modeling, offering advantages in data analysis. We also observed better signal-to-noise and narrower ^1^H linewidths in ^1^H-^13^C TROSY spectra collected on selectively labeled samples compared to its uniformly labeled counterpart. To take advantage of these benefits, our atom-selectively labeled [2-^13^C, 7-^15^N]-ATP was also used to measure ^13^C R_2_ relaxation rates and steady-state ^13^C{^1^H} hNOE values in HBV ε. These spin relaxation measurements provide a starting point to a robust understanding of HBV ε dynamics and suggest that residue A13 has increased flexibility whereas A21 may have R_ex_ contributions.

## Supplementary Information

Below is the link to the electronic supplementary material.

Supplementary file1 (PDF 827 KB)

## Data Availability

All results generated in this study are included in this published article and its Supplementary Materials.
