# Supplementary material for: Quantifying the effects of long-range 13C-13C dipolar coupling on measured relaxation rates in RNA
Source: J Biomol NMR. 2021 Apr 29;75(4):203–11. doi: 10.1007/s10858-021-00368-8 (PMC8131303; doi:10.1007/s10858-021-00368-8)
Supplement: Supplementary file 1 — Supplementary file1 (PDF 827 KB) [file 10858_2021_368_MOESM1_ESM.pdf]

Supporting Material

to

Quantifying the effects of long-range ^{13}C-^{13}C dipolar coupling on measured relaxation rates in RNA

Lukasz T. Olenginski and Theodore K. Dayie*

Center for Biomolecular Structure and Organization, Department of Chemistry and Biochemistry, University of Maryland, College Park, MD 20742, United States.

* To whom correspondence should be addressed. Tel: 301-405-3165; Email: dayie@umd.edu

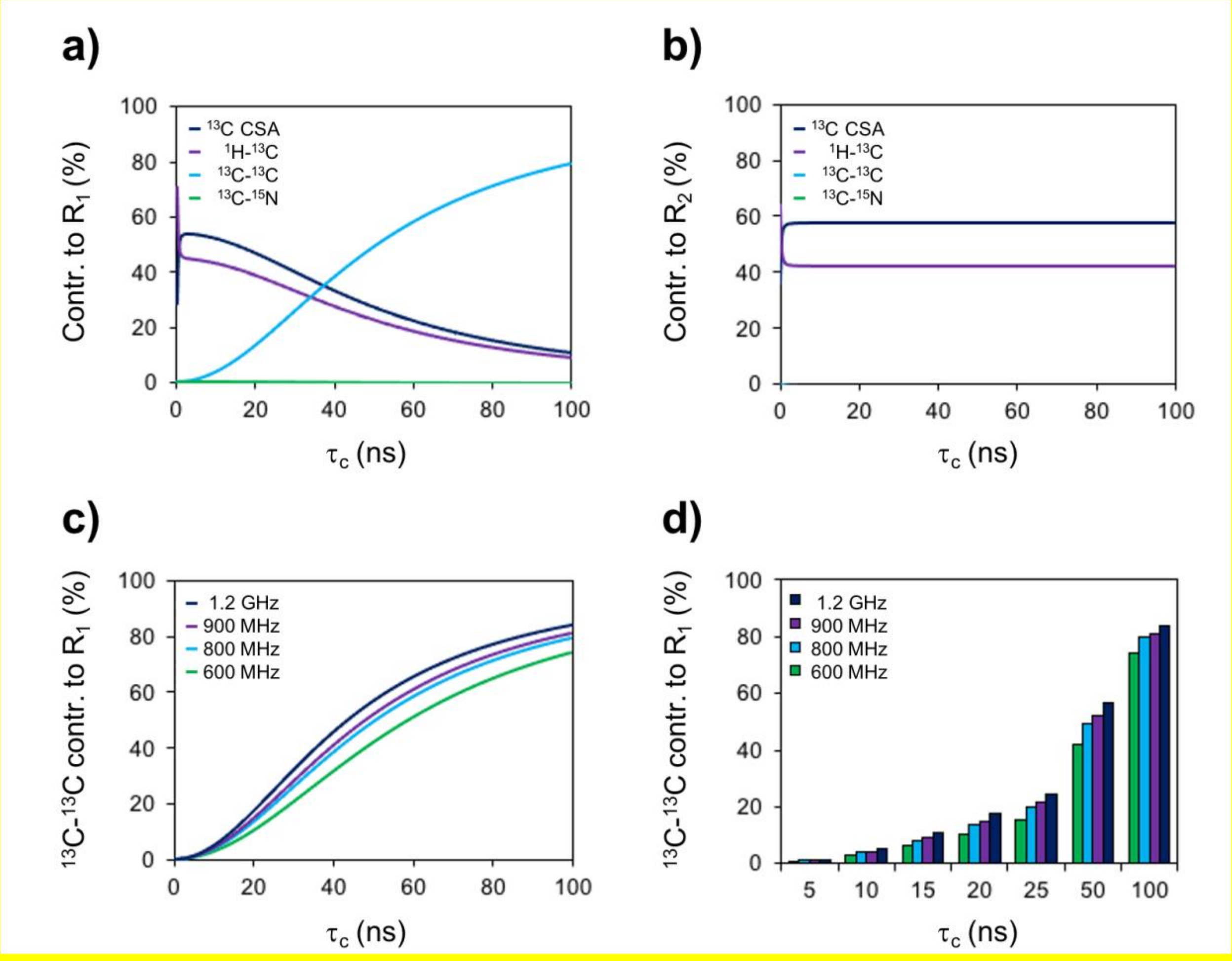

Supplementary Fig. S1 Quantifying the contributions to adenosine $R_{1,C2}$ and $R_{2,C2}$ relaxation rates. **a** Simulated ^{13}C CSA, ^{1}H-^{13}C, ^{13}C-^{13}C, and ^{13}C-^{15}N dipolar contributions (contr.) to adenosine $R_{1,C2}$ rates at 800 MHz. **b** Simulated ^{13}C CSA, ^{1}H-^{13}C, ^{13}C-^{13}C, and ^{13}C-^{15}N dipolar contributions (contr.) to adenosine $R_{2,C2}$ rates at 800 MHz. Here, the ^{13}C CSA and dipolar contributions are defined as [100*(^{13}C2 CSA/$R_{1/2,C2}$)] and [100*(^{1}H-^{13}C dipolar/$R_{1/2,C2}$)], [100*(^{13}C-^{13}C dipolar/$R_{1/2,C2}$)], or [100*(^{13}C-^{15}N dipolar/$R_{1,C2}$)], respectively, where the CSA and dipolar terms are those found in Eqs. 3 and 5. $R_{1/2,C2}$ refers to either $R_{1,C2}$ or $R_{2,C2}$. ^{13}C-^{13}C and ^{13}C-^{15}N dipolar contributions to $R_{2,C2}$ are so negligible that they are unobserved given the plotted scale. **c** Simulated ^{13}C-^{13}C dipolar contributions to adenosine $R_{1,C2}$ at increasing magnetic field strength. **d** Same as in **c** but highlighting specific RNA sizes (measured by correlation times (τ_C)) to correspond with those mentioned in the text. All simulations assume isotropic tumbling and were carried out with increasing τ_C. Solution NMR derived CSA values (σ_{11} = 89, σ_{22} = 15, σ_{33} = -104)[34] and an aromatic CH bond length of 1.104 Å[35] were used. Our simulations suggest that the ^{13}C CSA and ^{1}H-^{13}C dipolar contributions dominate adenosine $R_{1,C2}$ (for RNAs with τ_C < 40 ns) and $R_{2,C2}$ (for all RNAs) relaxation. However, ^{13}C-^{13}C dipolar interactions dominate adenosine $R_{1,C2}$ relaxation for RNAs with a τ_C > 40 ns, especially at higher magnetic fields.

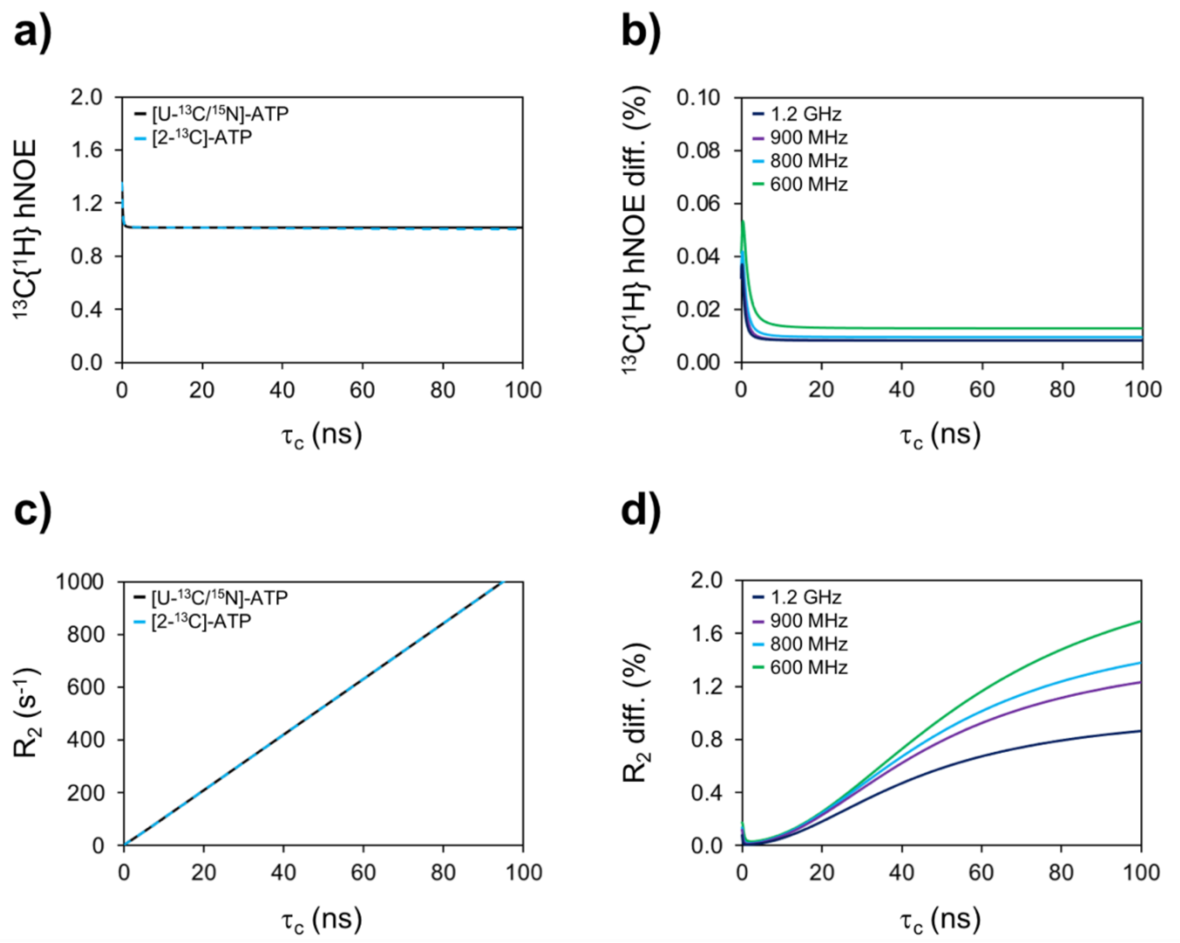

Supplementary Fig. S2 Adenosine C2 relaxation simulations in [U-^{13}C/^{15}N]-ATP or [2-^{13}C, 7-^{15}N]-ATP labeled RNAs. **a** Simulated steady-state ^{13}C{^{1}H} hNOE values. **b** Simulated steady-state ^{13}C{^{1}H} hNOE percent difference (diff.) [100*(hNOE$_{(uniform)}$ – hNOE$_{(selective)}$)/hNOE$_{(uniform)}$]. **c** Simulated adenosine $R_{2,C2}$ rates. **d** Simulated $R_{2,C2}$ percent difference [100*($R_{1,C2(uniform)}$ – $R_{1,C2(selective)}$)/$R_{1,C2(uniform)}$]. All simulations were carried out assuming isotropic tumbling and with increasing magnetic field strengths and overall correlation times (τ_C) except those in **a** and **c** which were at 800 MHz. Solution NMR derived CSA values (σ_{11} = 89, σ_{22} = 15, σ_{33} = -104)[1] and an aromatic CH bond length of 1.104 Å[2] were used. There is no significant differences between the simulated $R_{2,C2}$ rate or steady-state ^{13}C{^{1}H} hNOE between [U-^{13}C/^{15}N]-ATP or [2-^{13}C]-ATP labeled RNAs. Therefore, dipolar interactions to adenosine C2 do not significantly contribute to these relaxation parameters.

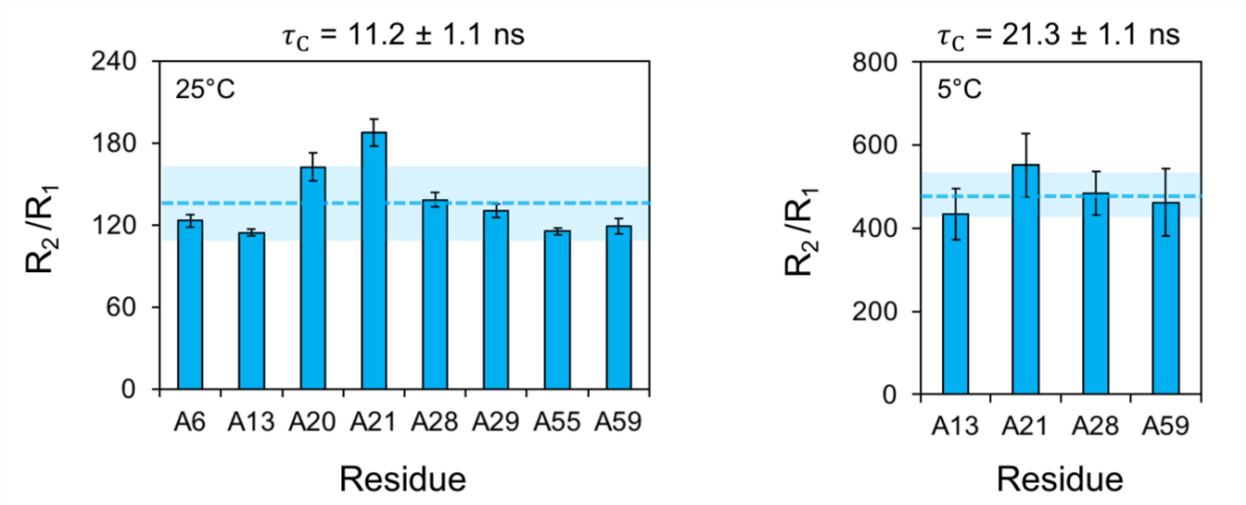

Supplementary Fig. S3 τ_C estimates from R_2/R_1 ratios derived from experimental adenosine C2 relaxation measurements for [2-^{13}C, 7-^{15}N]-ATP labeled HBV ε at 800 MHz and 5 and 25°C. R_2 was calculated from $R_{1\rho,C2}$ using Eqs. 6 and 7 (see main text). The τ_C of HBV ε was estimated from the R_2/R_1 by the following relation[3,4]: $\tau_c \sim \frac{1}{2\omega_C}\sqrt{6\frac{R_2}{R_1}-7}$ where ω_C is the ^{13}C Larmor frequency. Based on experimental data, HBV ε has a τ_C of 11.2 ± 1.1 ns and 21.3 ± 1.1 ns at 25 and 5°C, respectively, in agreement with those obtained with more detailed analysis using ROTDIF[5] (data not shown). Error bars represent ± standard deviation (s.d.) and the mean relaxation parameters are shown with dashed lines with a shaded box representing ± s.d. above and below the mean.

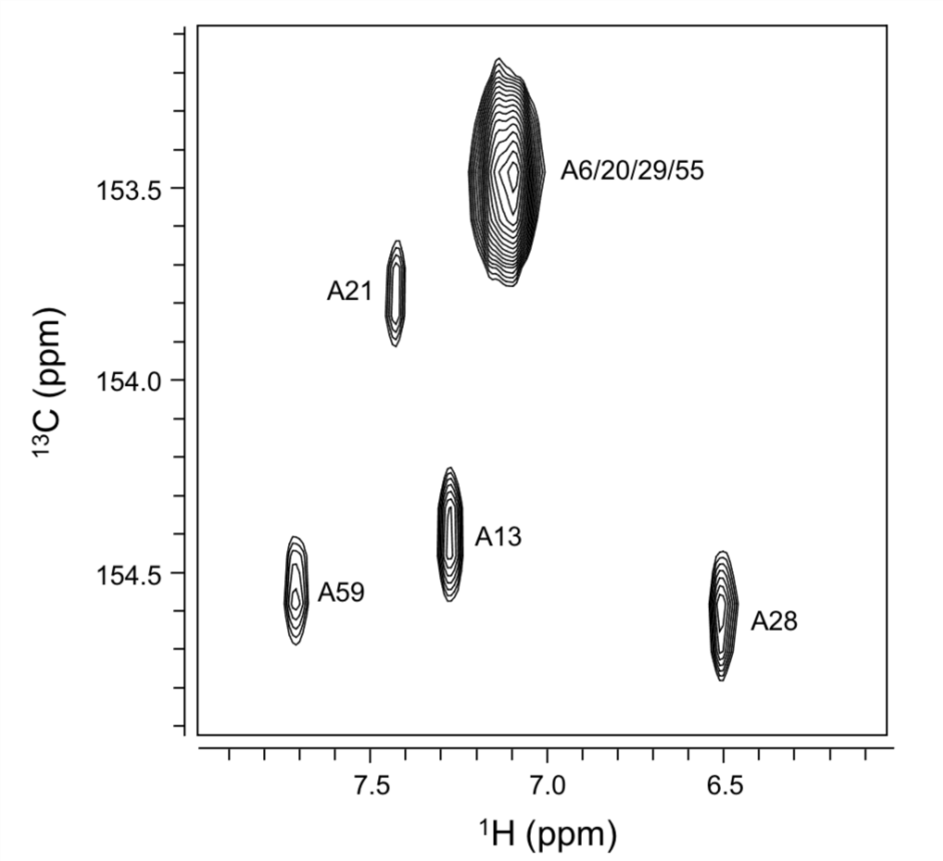

Supplementary Fig. S4 ^{1}H-^{13}C TROSY spectrum at lower temperature. The spectra shown was collected for [2-^{13}C, 7-^{15}N]-ATP labeled HBV ε at 800 MHz and 5°C. Compared to spectra collected at 25°C (see Supplementary Fig. S6), those at 5°C were collected with a reduced sweep-width and time-domain points with an increased number of scans to maximize signal-to-noise. Accordingly, only 4 of the 8 adenosine C2-H2 resonances were resolved.

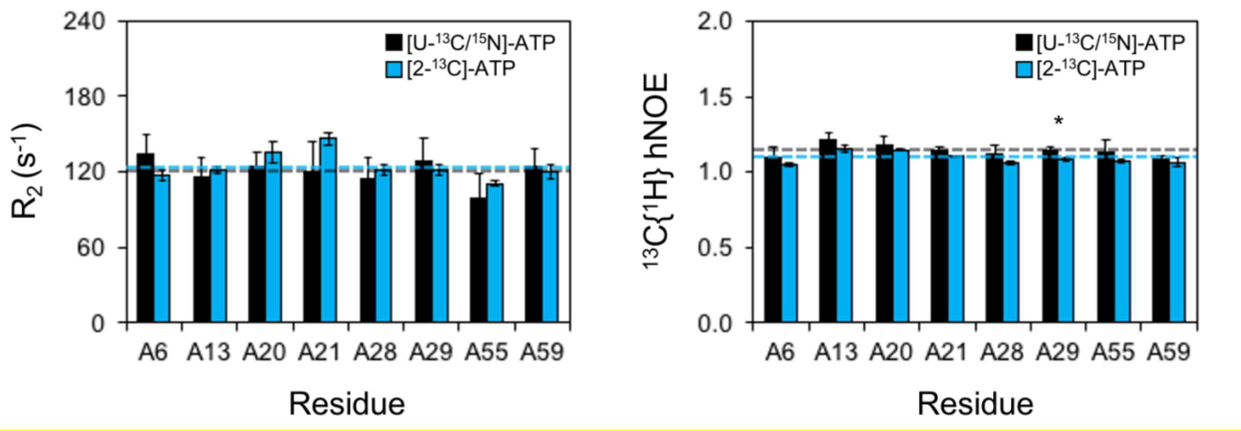

Supplementary Fig. S5 Experimental adenosine $R_{2,C2}$ and steady-state ^{13}C{^{1}H} hNOE measurements in [U-^{13}C/^{15}N]-ATP or [2-^{13}C, 7-^{15}N]-ATP labeled HBV ε RNA. **a** Adenosine $R_{1,C2(uniform)}$ and $R_{1,C2(selective)}$ rate (left) and $hNOE_{(uniform)}$ and $hNOE_{(selective)}$ (right) measurements in HBV ε at 800 MHz and 25°C. Mean rates are shown with dashed lines and error bars represent ± s.d.. Experimental $R_{2,C2(uniform)}$ and $R_{2,C2(selective)}$ rates do not differ significantly (values are within experimental error). Experimental $hNOE_{(uniform)}$ and $hNOE_{(selective)}$ values also do not differ significantly, except A29 residues (designated *). Taken together, our simulations and experimental measurements suggest that there is no significant difference in adenosine $R_{2,C2}$ rates and steady-state ^{13}C{^{1}H} hNOE values in uniformly and selectively labeled samples.

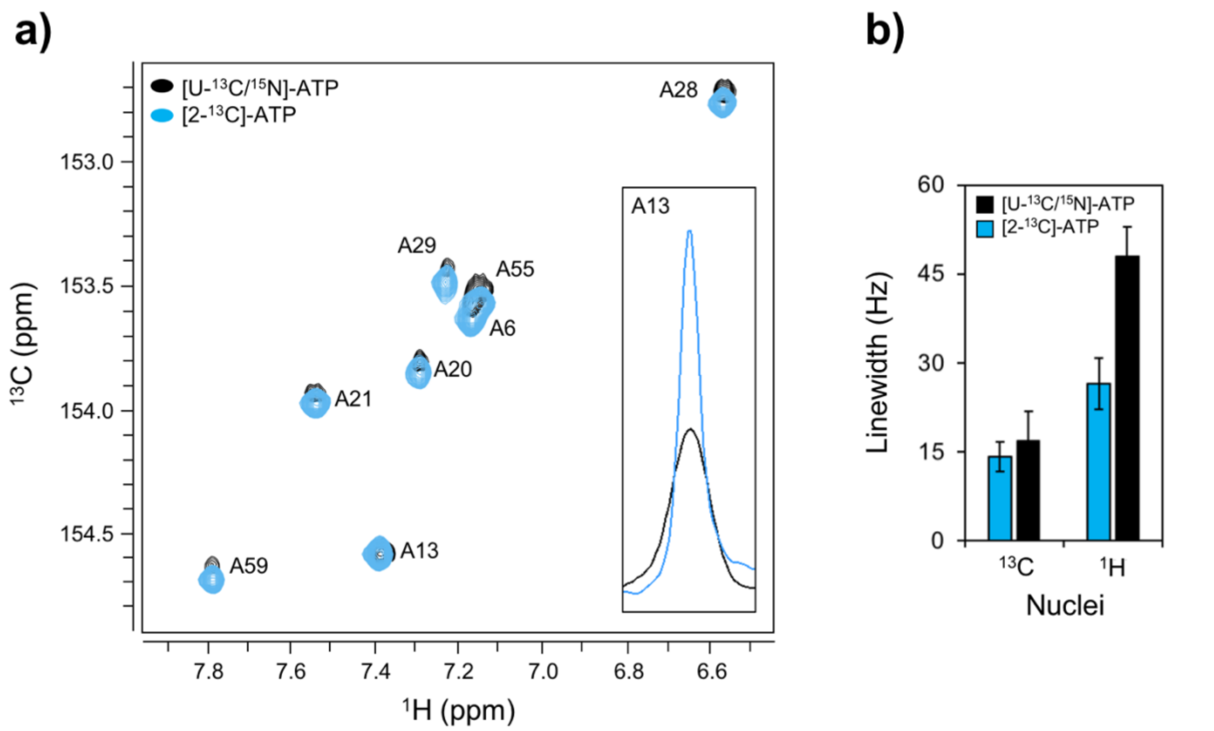

Supplementary Fig. S6 Comparison of NMR spectral properties of [U-^{13}C/^{15}N]-ATP or [2-^{13}C, 7-^{15}N]-ATP labeled HBV ε. **a** ^{1}H-^{13}C TROSY spectrum for HBV ε adenosine H2-C2 resonances with inset showing ~2-fold improvement in signal-to-noise from selective [2-^{13}C, 7-^{15}N]-ATP labeling for a ^{1}H slice of residue A13. The spectra shown was collected at 800 MHz and 25°C **b** Comparison of TROSY linewidths of [U-^{13}C/^{15}N]-ATP or [2-^{13}C, 7-^{15}N]-ATP labeled HBV ε. The bar plot represents average linewidths (both ^{1}H and ^{13}C) for all 8 adenosine H2-C2 resonances, calculated (using a Lorentzian fit using TopSpin 4.0) from the spectra shown in **a**. Error bars represent ± s.d. [U-^{13}C/^{15}N]-ATP and [2-^{13}C, 7-^{15}N]-ATP labeled NMR samples were of equal concentration and spectra were recorded with an equivalent number of scans and time-domain points. Selective [2-^{13}C, 7-^{15}N]-ATP labeling leads to better signal-to-noise and narrower ^{1}H linewidths while ^{13}C linewidths remained unchanged.

References

1. Ying, J., Grishaev, A., Bryce, D. L. & Bax, A. Chemical shift tensors of protonated base carbons in helical RNA and DNA from NMR relaxation and liquid crystal measurements. *J. Am. Chem. Soc.* **128**, 11443–11454 (2006).
2. Fiala, R., Czernek, J. & Sklenář, V. Transverse relaxation optimized triple-resonance NMR experiments for nucleic acids. *J. Biomol. NMR* **16**, 291–302 (2000).
3. Fushman, D., Weisemann, R., Thüring, H. & Rüterjans, H. Backbone dynamics of ribonuclease T1 and its complex with 2′GMP studied by two-dimensional heteronuclear NMR spectroscopy. *J. Biomol. NMR* **4**, 61–78 (1994).
4. Thakur, C. S., Sama, J. N., Jackson, M. E., Chen, B. & Dayie, T. K. Selective ^{13}C labeling of nucleotides for large RNA NMR spectroscopy using an E. coli strain disabled in the TCA cycle. *J. Biomol. NMR* **48**, 179–192 (2010).
5. Berlin, K., Longhini, A., Dayie, T. K. & Fushman, D. Deriving quantitative dynamics information for proteins and RNAs using ROTDIF with a graphical user interface. *J. Biomol. NMR* **57**, 333–352 (2013).
